# Exogenous dsRNA made accessible to Dicer by two eukaryotic RNA-dependent RNA polymerases in *Paramecium tetraurelia*

**DOI:** 10.1038/s42003-025-09443-4

**Published:** 2026-01-08

**Authors:** Marcello Pirritano, Johannes Buescher, Pauline Staubach, Thorsten Tacken, Yulia Yakovleva, Mark Sabura, Kristela Shehu, Sören Franzenburg, Marc Schneider, Martin Simon

**Affiliations:** 1 Molecular Cell Biology and Microbiology, Faculty for Mathematics and Natural Sciences, Wuppertal University, Wuppertal, Germany; 2 Department of Pharmacy Biopharmaceutics and Pharmaceutical Technology, Saarland University, Saarbrücken, Germany; 3 PharmaScienceHub (PSH), Saarbrücken, Germany; 4 INM—Leibniz Institute for New Materials, Saarbrücken, Germany; 5 Competence Centre for Genomic Analysis, CCGA, Kiel, Germany; 6 Present Address: Institute of Medical Virology, Justus Liebig University Giessen, Giessen, Germany

**Keywords:** RNAi, Nanoparticles

## Abstract

Discrimination of self from non-self RNA is a critical requirement for any cell to respond to infections and to maintain cellular integrity. We report novel functions for two RNA-dependent RNA polymerases (RDRs) in *Paramecium*. In RNAinterference (RNAi), RDRs are normally involved in the production of large amounts of secondary small interfering RNAs (siRNAs). To characterize the function of RDRs in context of exogenous RNA recognition, we developed a novel double-stranded RNA (dsRNA) application system using dextran nanoparticles to deliver heteroduplex dsRNA to cells as food particles, mimicking the natural phagosomal entry. Small RNA sequencing allows to dissect siRNAs produced from exogenous RNA or RDR transcripts. Contrary to expectations, our data show that Dicer is unable to directly cleave exogenous dsRNA while two RDRs, RDR1 and RDR2, are required for the initial steps of dsRNA-induced RNAi. Paradoxically, these two RDRs must replicate dsRNA before Dicer cleavage. This system works efficiently also with exogenous single-stranded RNA (ssRNA), although RDR2 is dispensable for ssRNA conversion. The function of RDRs is in contrast to that in animals, plants and fungi and extends the functional diversity of these polymerases as RDR-associated complexes appear to control the entry of food RNA into the RNAi machinery.

## Abstract (web summary)

Phagosomal delivery of heteroduplex RNA sheds light into the initial recognition of exogenous RNAs and entry into the RNA interference pathway.

## Introduction

Any cell needs to have molecular mechanisms to recognize foreign RNA from pathogens, while endogenous RNA needs to be tolerated. RNA in general is a major menace to genome stability, and has led to a variety of mechanisms for its degradation or introduction into the RNAi pathway^1^. Double-stranded RNA (dsRNA) in particular was shown to trigger various mechanisms. In mammals, RigI-like receptors, PKRs and Toll-like receptors specifically recognize dsRNA and activate diverse immune responses^2,3^. Non-vertebrates, in contrast, use RNA interference (RNAi) for antiviral defense, which involves the production of siRNAs to inactivate viral genomes and mRNAs. It has long been thought that mammals do not use antiviral RNAi, but a growing body of data supports the existence of RNAi in mammals; however, siRNA generation seems to be in competition with the interferon system^4^.

Dicer activity is key to determining whether dsRNA triggers RNAi^5^. In nematodes, for example, exogenous dsRNA is rapidly cleaved by endogenous Dicer into functional siRNA^6^. Also in mammals, Dicer dissects the fate of dsRNA. Although canonical Dicer appears to be unable to cleave exogenous dsRNA, an oocyte-specific isoform lacking the helicase domain has increased activity on dsRNA^7^. Even more intriguingly, canonical Dicer in mammals has been implicated as a central mediator of antiviral responses by mediating a crosstalk to PKR and NF-kB^8^.

Here, we analyze the cellular response to exogenous dsRNA in the ciliate *Paramecium*, in which RNAi can be easily induced by feeding dsRNA (reviewed in ref. ^9^). We provide evidence that this dsRNA is not the trigger molecule and is not processed directly by Dicer. Instead, exogenous dsRNA is first recognized by RNA-dependent RNA polymerases (RDR).

Eukaryotic RDRs are described for fungi, plants, animals, and also protists. In all these kingdoms except protists, the literature describes their general function in a process called “RNAi amplification”, which means the accumulation of large amounts of 2° (secondary) siRNAs in response to a 1° (primary) siRNA that is cut by Dicer from an initial trigger molecule. RNAi amplification as used in literature comprises two aspects: RDR activity on mRNA and the production of a large amount of 2°siRNAs as a result of fewer 1° trigger molecules^10^. This is demonstrated for yeast^11,12^, plants^13^, and also for *C. elegans*^14^. However, the nematode might not be representative for the animal kingdom, as RNAi in animals has been described to work without RDR amplification. Recent data suggest the existence of RDRs in animal genomes, but apparently with functions outside of siRNA amplification^15^. In contrast to detailed knowledge on RDRs in fungi, plants, and nematodes, functional analyses of protist RDRs are rare.

Asking for a role of RNAi amplification in unicellular species, here we describe the function of two distinct RDRs in *Paramecium* in response to feeding of dsRNA. This particular method is an exciting mechanism to rapidly inactivate gene expression by simply feeding dsRNA-expressing *E. coli*^16^ as described before in *C. elegans*^17^. Also similar to the nematode, 2° siRNAs have been demonstrated to occur, produced from the entire target mRNA^18^. While these appear to be Dicer independent, Dcr1 has been shown to be necessary for 1° siRNA production from dsRNA^19^: while the mechanistic role of RDR1 and RDR2 in 1° siRNA accumulation remains unclear^20^.

## Results

### 1° and 2° siRNAs in *Paramecium* are predominantly 5′-monophosphorylated

We first evaluated the biochemical properties of siRNAs. This is necessary since RDR-dependent amplification mechanism are diverse: in plants, they produce long dsRNA and subsequent Dicer cleavage creates double stranded siRNAs carrying a 5′-mono-phosphate (5′-P)^21^, whereas 2° siRNAs carrying a 5′-tri-phosphate (5′-PPP) in *C. elegans* as a result from de novo transcription by RDR^22^.

**Fig. 1:**
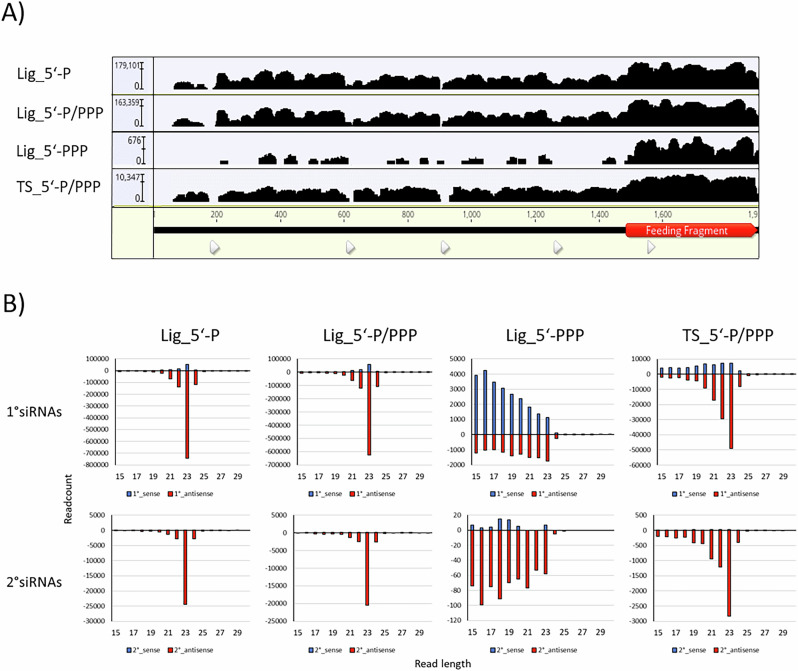
Biochemical analysis of feeding-associated siRNAs. **A** Coverage of 23 nt siRNA reads produced from dsRNA feeding against the *ND169* gene. The feeding fragment is highlighted in red (1^∘^ siRNAs). siRNAs outside of the feeding fragment region are 2^∘^ siRNAs. Libraries enriching RNAs for individual 5′-phosphorylation status are: P mono-phosphorylation, PPP  -tri-phosphorylation. Lig - ligation-based library preparation, TS -  template switch based library. Note the logarithmic scale. **B** Read length distribution of 1^∘^ (top row) and 2^∘^ (bottom row) small RNAs derived by dsRNA feeding present in libraries of different types (Lig for ligation-based and TS for Template switch-based) enriching for different 5′ -phosphorylation states (compare “Methods” section).

For *Paramecium*, it must be noted in advance, that 2° siRNAs have been reported which map to mRNA outside of the feeding region^18^. In contrast to other species, these 2° siRNAs are not more abundant than 1° siRNA. While 1° siRNAs have been biochemically characterized by Northern blot to be predominantly 5’-mono-phosphorylated^20^, this former analysis does not allow to dissect minor sRNA abundances with different phosphostatus nor the detection of the low-abundant 2° siRNAs. To clarify here whether RDR-associated siRNAs produce any other than 5′-P siRNAs, we treated RNA samples from RNAi against the *ND169* reporter gene either with Cap-Clip pyrophosphatase (converting any phosphorylation in ligatable 5′-P), or by Terminator™ digestion with subsequent Cap-Clip treatment (digest of 5′-P RNA with subsequent conversion of any remaining RNAs into 5′-P RNA). Both treatments were then subjected to classical, 5′-P-specific ligation-based library preparation. In addition, a 5′-phosphorylation independent template switch-based library preparation was carried out. Figure 1A shows siRNA coverage of the *ND169* gene for all treated RNAs, highlighting the fragment used for dsRNA synthesis (corresponding to 1° siRNAs) with 2° siRNAs outside of the highlighted area. Comparing the different treatments with the 5′-P specific library, we cannot identify any differences: abundant 1° siRNAs in the dsRNA region and a low coverage of 2° siRNAs outside these regions in all samples (ratio 1°/2° approx. 2–5%) (Supplementary Fig. 1B). Please note the low read number in the Terminator™-treated sample. This library was almost invisible during gel extraction, indicating that only remnants of RNA were ligated which is supported by the read length distribution (Fig. 1B). Therefore, we can rule out that we miss a high number of 2° siRNAs by 5′-P dependent ligation procedures and that 2° siRNAs are much less abundant than 1° in *Paramecium* (Supplementary Fig. 1B).

### 1° siRNAs are a mixture of applied dsRNA and RDR products

**Fig. 2:**
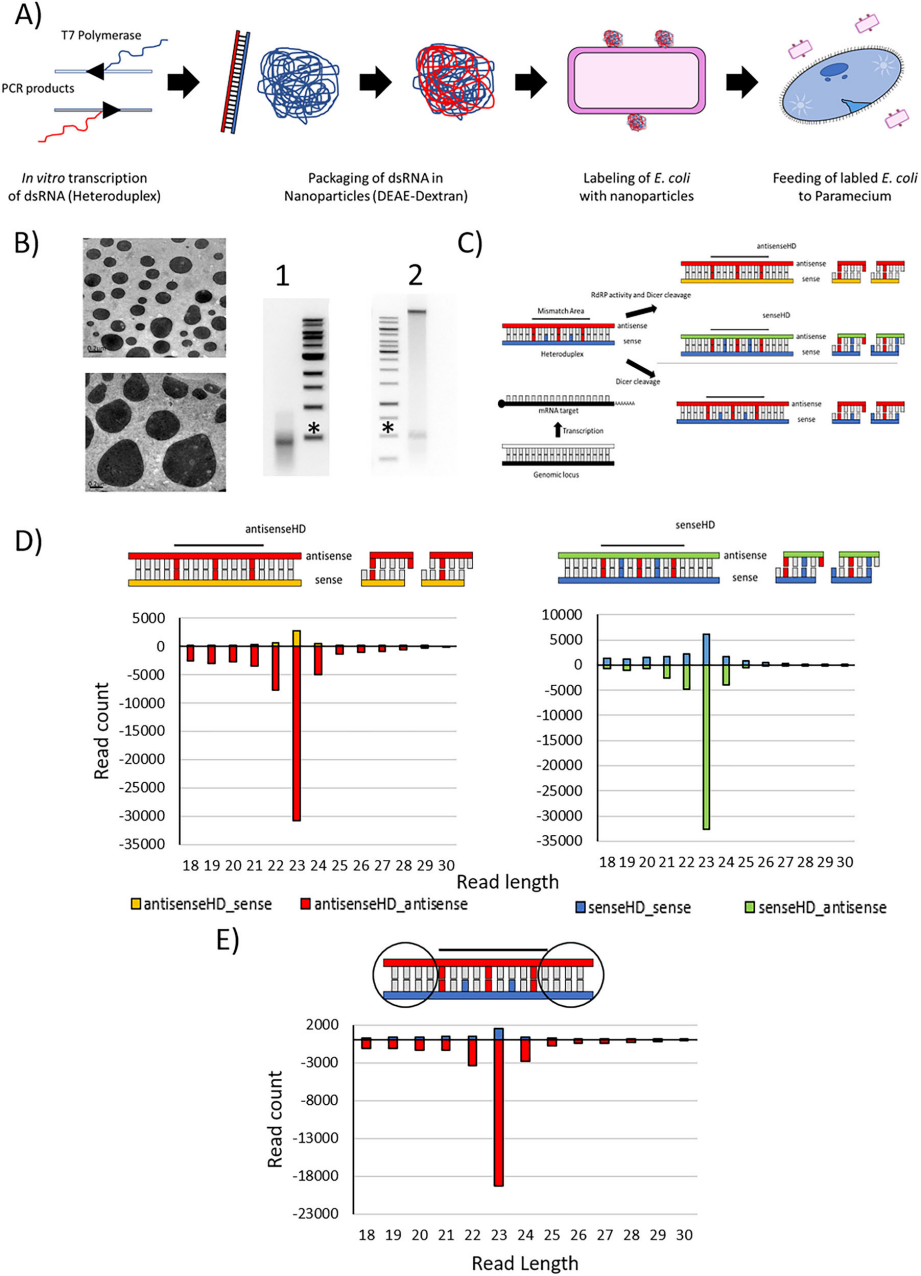
Application of heteroduplex dsRNA to analyze 1^∘^siRNA production. **A** Workflow of heteroduplex application from in vitro transcription, nanoparticles assembly, labeling of *E. coli* and application of labeled bacteria to *P. tetraurelia*. **B** Left: Transmission electron microscopic image of nanoparticles. Scale bars are 0.2 μm. Right: Native Gel electrophoretic analysis of annealed dsRNA before nanoparticle formation (lane 1) and after particle formation and adsorption to *E. coli* (lane 2). RNA in lane 2 was re-isolated from living bacteria. The high molecular band corresponds to the bacterial gDNA. Asterisks indicate 500bp Marker bands. **C** Scheme of heteroduplex strands (blue and red) in association to the endogenous target. Mismatches between heteroduplex strands, as well as the endogenous sequences are highlighted. RDR activity on the heteroduplex leads to new strands (orange, green). Lack of RDR activity leads to generation of siRNAs solely based on original heteroduplex strands. **D** Read length distribution of reads mapping to the four possible heteroduplex dsRNA derived strands. Read count of reads corresponding to sense and antisense strands of the original strands (blue and red, respectively) and to the RDR-dependent products (green and orange) are shown. **E** Read length distribution of reads mapping to the area of the heteroduplex without mismatches to the endogenous sequence. Due to the absence of mismatches, RDR-produced products can not be distinguished from exogenously applied strands. Read count of reads in sense (blue) and antisense (red) orientation to the endogenous sequence are shown.

In order to elucidate the role of RDR1 and RDR2, we decided to apply a specific heteroduplex dsRNA in vivo (specific details regarding the heteroduplex dsRNA design can be found in the Methods section). Since direct production of heteroduplex dsRNA in *E. coli* is impossible due to rapid recombination of the two nearly identical parts in the plasmid, we annealed single-stranded in vitro transcripts and incorporated them into dextran nanoparticles (Fig. 2A, B). To guarantee cell entry through the natural phagosomal pathway, the dextran particles were adsorped to *E. coli* cell surface and then fed to paramecia (Fig. 2A). Successful re-isolation of intact dsRNA from *E. coli* demonstrated the stability of the trigger molecules (Fig. 2B). After feeding the manipulated *E. coli* to paramecia, small RNAs were sequenced and mapped to the individual template strands of the heteroduplex sequence containing the mismatches. The heteroduplex dsRNA is designed with mismatches (i) between the two strands and (ii) with the target mRNA. This allows dissection of which strand a siRNA is derived from, one of the original applied strands (blue/red, labeled as senseHD_sense and antisenseHD_antisense describing the original strand of the heteroduplex and the orientation of the smallRNAs, respectively), their RDR transcripts (green, yellow, labeled senseHD_antisense and antisenseHD_sense, following the same naming convention), or from the target mRNA (black/white) (Fig. 2C and Supplementary Fig. 2A, see Supplementary Table 1 for the sequences used and in depth information about mismatch distribution.).

Figure 2D shows that we can detect siRNAs not only from the exogenously applied strands (blue and red) but also from RDR transcripts of both exogenous strands (green and yellow, compare schematic overview in Fig. 2C). The experiments were repeated four times with almost identical results (Supplementary Fig. 2B, C). We conclude that 1° siRNAs in *Paramecium* are a mixture of endogenous RDR products and exogenously applied RNA. RDR products are not in excess, which argues against massive amplification. We can also detect a very low number of antisense reads originating from mRNA, corresponding to 2° siRNAs (Supplementary Fig. 2B).

Length distributions of siRNAs show a 23 nt peak with a preference for antisense siRNAs, regardless of whether they originate from the original dsRNA (red/blue) or from RDR products (green/orange) (Fig. 2D and Supplementary Fig. 2C). To evaluate whether this antisense preference could be due to the distribution of mismatches, we performed an experiment with a switched heteroduplex, in which the distribution of mismatches on sense and antisense strand of the dsRNA is reversed. Supplementary Fig. 3 shows that antisense siRNAs are also more abundant than sense siRNAs in this RNA. We conclude that stabilization of antisense siRNAs occurs independently of the origin (exogenous or RDR product) of the strand.

Our design of the applied heteroduplex RNA also includes two flanking regions 5′ and 3′ of the central heteroduplex area that do not include any mismatches to determine whether mismatches in the central regions cause any conflicts with intracellular molecular recognition in this species. Figure 2E (and Supplementary Fig. 2D) shows that this area also produces 23 nt siRNAs predominantly of the antisense strand. Please note that the coloring reads from the non-mismatch area (blue/red) is due to the mapping to the endogenous sequence: this does not imply that these do not contain RDR products but we simply cannot dissect these due to the sequence.

### Sense siRNAs carry non-templated uridines

To gain a deeper understanding of the accumulation of antisense RNAs, we screened the sRNAs resulting from heteroduplex feeding for sequence characteristics. Suppl. Fig. 4A shows that we cannot identify a preference for nucleotides in any siRNA derived from the heteroduplex, regardless of investigated strand. This distinguishes feeding associated small RNAs from other sRNA produced by Dicer1 in *Paramecium*, as e.g., transgene-induced siRNAs show a 5′-U preference^23^. The sequence logo analysis of normal dsRNA feeding also did not reveal any nucleotide preferences, so we can rule out putative influences of the mismatches present in the heteroduplex dsRNA in the analysis (Supplementary Fig 4 B). Classical non-heteroduplex dsRNA feeding allows for an analysis of overlapping reads. Supplementary Fig. 4B shows a peak for 21 nt overlap probability which would fit to 23 nt Dicer cuts with 2 nt overlaps and this is consistent with previous reported that 1° siRNAs in *Paramecium* depend on Dcr1.^18,19^.

**Fig. 3:**
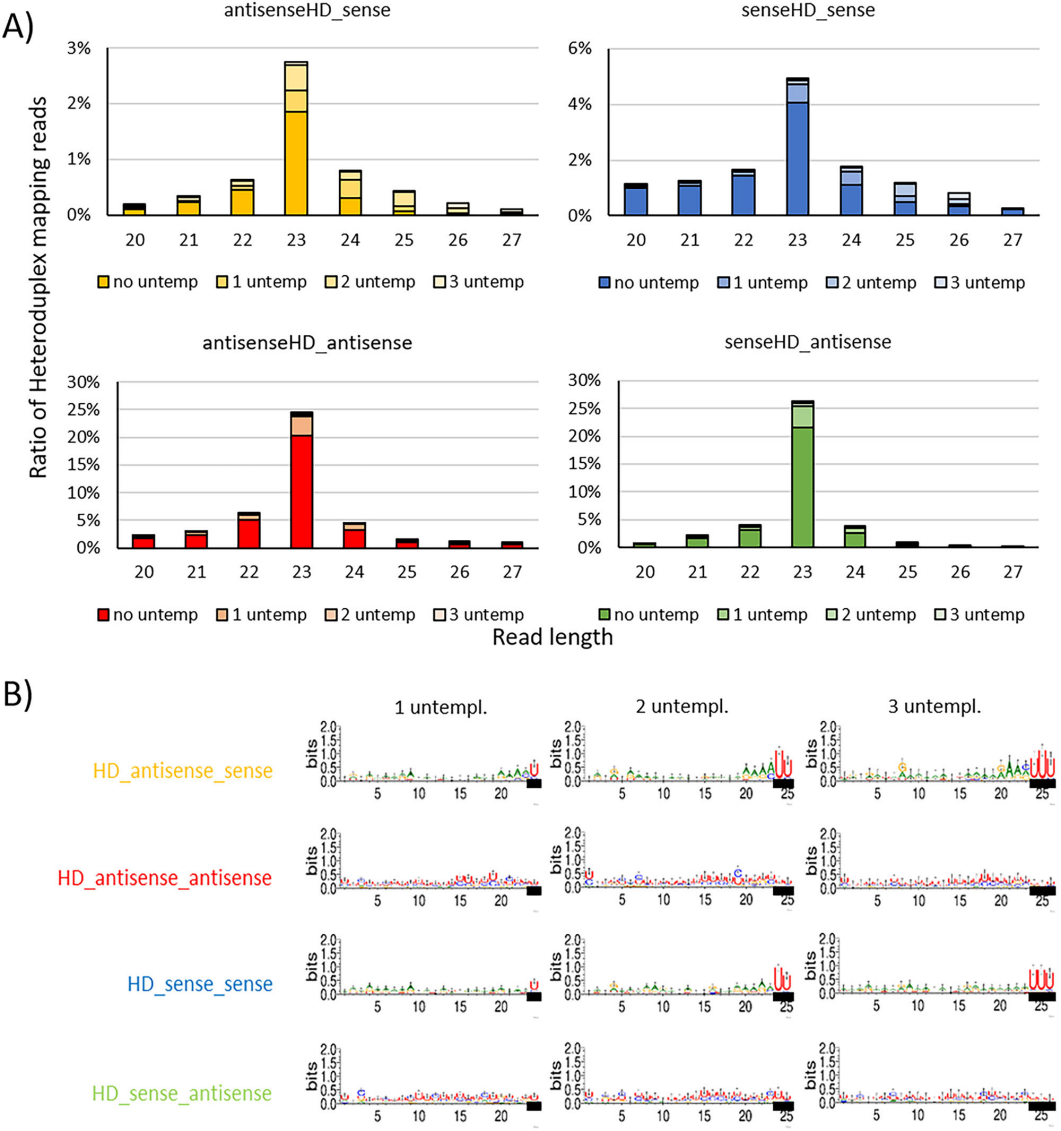
Distribution of non-templated nucleotides to different heteroduplex-derived 1^∘^siRNAs. Analysis of 3′-non-templated nucleotides in small RNAs. **A** Read length distribution of sRNAs mapping to the individual strands. Percent ratios of reads mapping to the four possible heteroduplex strands are displayed. Reads are catergoized in reads carrying no, one, two or three untemplated nucleotides. In relation to the original heteroduplex sequence, **B** sequence logos of reads carrying one, two or three untemplated nucleotides. Bases that are considered non-templated are labeled using a black bar.

Our analysis of non-templated nucleotides shows the presence of non-templated nucleotides on smallRNAs of all strands (Fig. 3A). An unbiased analysis of these non-templated nucleotides using a custom snakemake pipeline, including sequence logo analysis, was used to see whether these non-templated nucleotides are specific nucleotides that are added to smallRNAs or whether the non-templation consists of random nucleotides. Interestingly, non-templation of sense-orientated siRNAs consists of uridines added to the 23 nt long RNA, while non-templation of antisense-orientated smallRNAs seems to consist of random nucleotides without a specific signature (Fig. 3B). The uridinylation logo appears strongest when analyzing 23 nt long siRNAs that are elongated with non-templated nucleotides (i.e., a strong two-uridine-signal in the sequence logo of 25 nt long smallRNAs carry non-templation, corresponding to a 23 nt siRNA being elongated with two uridines). Since oligo-uridinylation of sRNAs is mostly associated with a targeting for degradation^24^, we hypothesize that sense siRNAs are targeted for degradation, explaining the observed antisense bias, while antisense siRNAs are stabilized in Ptiwi. Our data do not implicate any difference between the two types of antisense and sense strands, respectively, and suggest that all strands are processed in the very same manner, regardless whether they are produced by an RDR or are exogenously applied.

### Exogenous dsRNA cannot be cleaved by Dicer directly

**Fig. 4:**
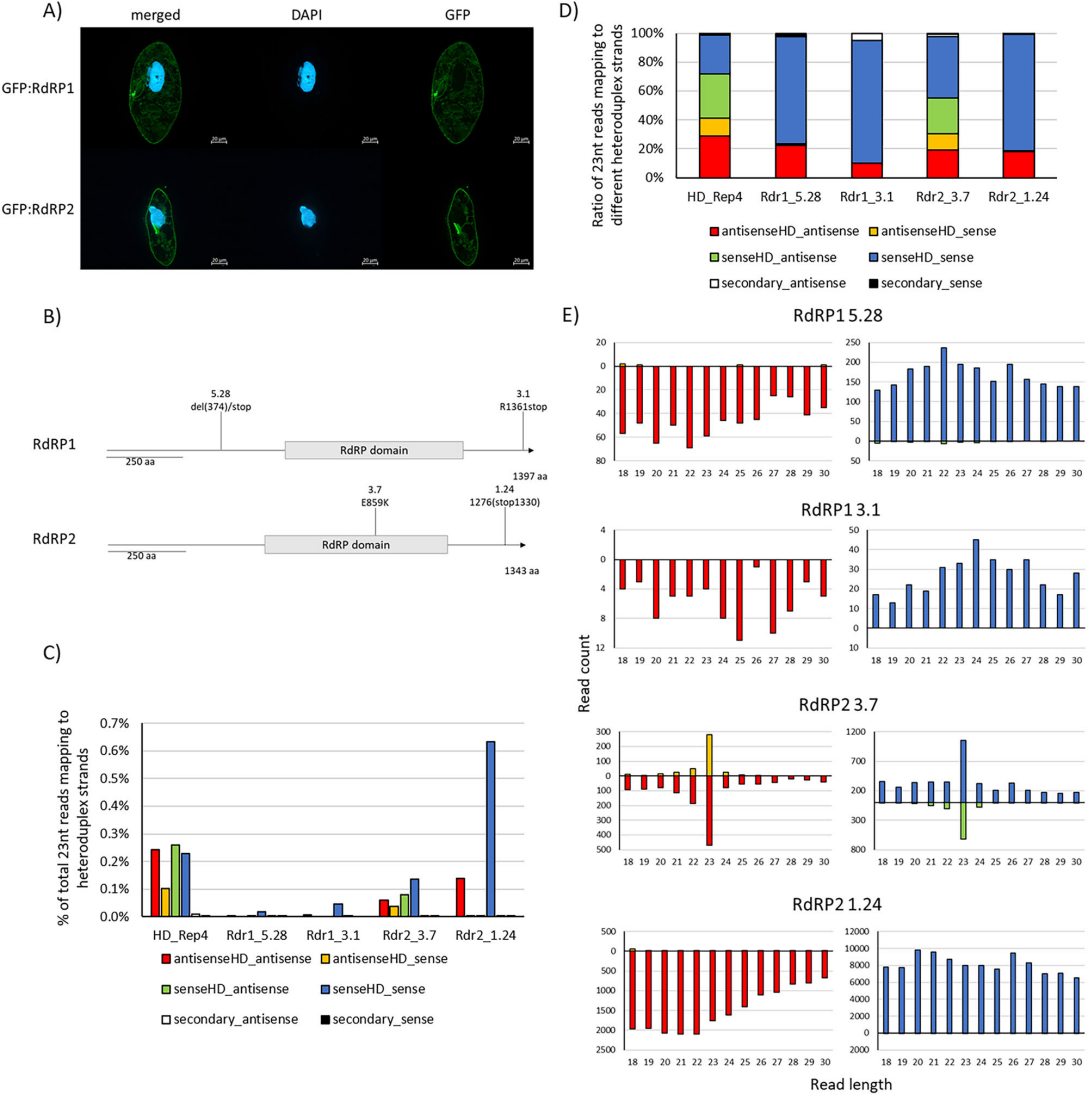
RDR-dependency of feeding-associated 1^∘^siRNAs. **A** Immunofluorescence localization of GFP:RDR1 and GFP:RDR2. Fluorescence signals generated by anti-GFP antibodies are shown. DAPI is used to counterstain the macronuclei. **B** Scheme of mutant cell lines used in upcoming experiments, including position and explanation of the carried mutations. **C** Abundance of 23 nt siRNA reads mapping to the different heteroduplex strands, color-coded according to the original strand as well as read orientation, in relation to total reads sequenced. Analyzed is the 23 nt siRNA abundance in wildtype as well as RDR-mutant strains. **D** Ratios of 23 nt siRNAs reads mapping to individual heteroduplex sequences, color-coded according to the original strand as well as read orientation, in relation to total number of 23 nt siRNA reads mapping to all heteroduplex strands. **E** Read length distribution of reads mapping to the different heteroduplex strands, color-coded according to the original strand as well as read orientation.

We proceeded with deeper investigations of the two RDRs. N-terminal GFP-fusion proteins of RDR1 and RDR2 co-localize in the cytosol (Fig. 4A). Supplementary Fig. 5 shows, in addition, that we cannot observe different localizations of both RDRs when dsRNA is applied to the cells, indicating that there is no special accumulation at e.g., vacuoles, when excessive dsRNA is ingested.

For functional analysis, we used previously produced *RDR* mutants deficient for dsRNA feeding^25^. These mutants result from a forward genetic screen of random mutagenesis and subsequent selection of dsRNA feeding deficient mutants by feeding of a lethal silencing construct^25^. This screen revealed two *RDR1* mutants have been shown to be null-alleles. *RDR2* revealed hypomorphic mutants only, suggesting that this is an essential gene^25^. Figure 4B shows the positions and type of mutations in *RDRs*. One mutant strain of each RDR shows truncated C-termini. Structural analysis of both RDRs suggests a highly similar structure of both with the C-termini directed outward, not being associated with the catalytic core (Supplementary Fig. 7). It seems tempting to speculate that these C-termini may be necessary for complex formation.

We fed the heteroduplex particles to these mutant strains and sequenced the resulting sRNAs. Figure 4C–E shows that no mutant line was able to produce wild type levels of 23 nt siRNAs. All mutants, except RDR2-3.7, are unable to produce any RDR products and importantly also do not show 23 nt siRNAs from applied dsRNA but instead only degradation products, indicating a complete lack of Dicer activity on exogenous dsRNA in *RDR* mutants.

We conclude that the applied dsRNA is not a Dicer substrate, but rather becomes accessible to cleavage only after conversion by RDRs into new sets of dsRNA. Both RDRs are necessary for this activity and the C-terminal region of both RDRs, which is devoid of any catalytic activity, appears to be crucial for this joint activity. In support of this, RDR2-3.7, which has a mutation in the catalytic domain but an intact C-terminus, exhibits reduced, yet still detectable, 23-nt siRNA accumulation.

To rule out the possibility that the results obtained from the heteroduplex region are compromised by mismatches, we also analyzed the non-mismatch regions of the dsRNA flanking the heteroduplex region on both sides. Suppl. Fig. 6A shows that the siRNAs resulting from this area are no more abundant than those from the mismatch area in wild-type cells. Since Supplementary Fig. 6B shows that the non-mismatch area siRNAs also depend on both RDRs, our data imply that both regions behave in the same way in our experiments, indicating that mismatches do not influence the cellular components. In other words, since *Paramecium* Dicer is also unable to cleave the non-mismatch area of the dsRNA, we can conclude that its inability to cleave the mismatch area of the same dsRNA molecule is not due to introduced mismatches.

### Applied ssRNA is efficiently converted into 23 nt siRNAs

**Fig. 5:**
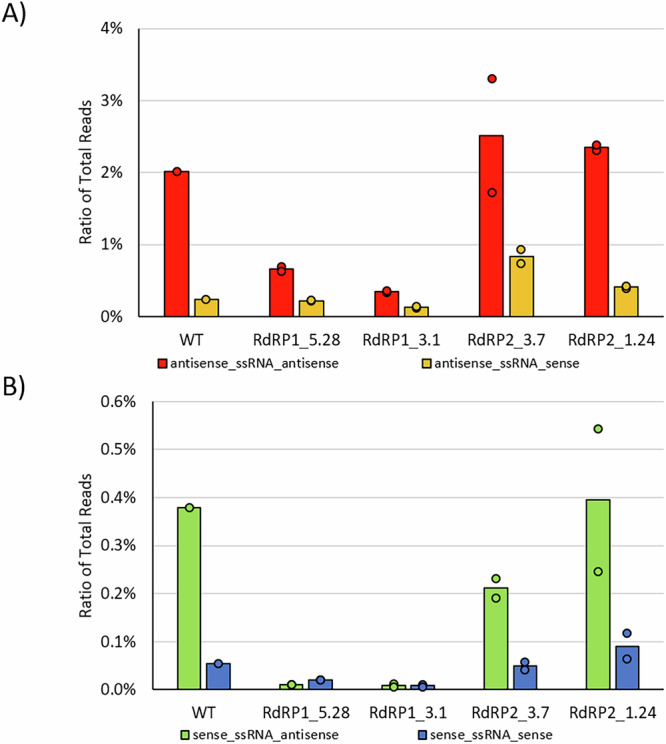
Abundance of 23nt siRNAs after ssRNA feeding in wildtype and *RDR*-mutants. Mapping statistics of sRNA sequencing of cultures undergoing single strand RNA (ssRNA) feeding: antisense-orientated ssRNA (**A**) and sense-orientated ssRNA (**B**) feeding was conducted. Displayed are the ratios of 23 nt siRNA reads mapping to the applied ssRNA. Bars represent the mean value of replicates, while points represent values of individual replicates.

If exogenous RNA was converted to dsRNA by RDRs, the next question was whether single-stranded RNA (ssRNA) could also trigger siRNA accumulation. Antisense 23 nt siRNAs, that map to bacterial transcripts, were previously found in *Paramecium*^18^. Figure 5 and Supplementary Fig. 8 show that both mismatch-containing sense and antisense ssRNA fed to *Paramecium*, can be efficiently converted into 23 nt siRNAs, which are again accumulated predominantly from the antisense strands. This process depends on RDR1 as both mutants reveal a lack of siRNAs. In contrast to the application of dsRNA, ssRNA feeding is apparently independent of RDR2, although siRNA profiles show differences in (i) more abundant sense siRNAs and (ii) less pronounced 23 nt peaks for the R2-3.7 mutant (Supplementary Fig. 8).

## Discussion

**Fig. 6:**
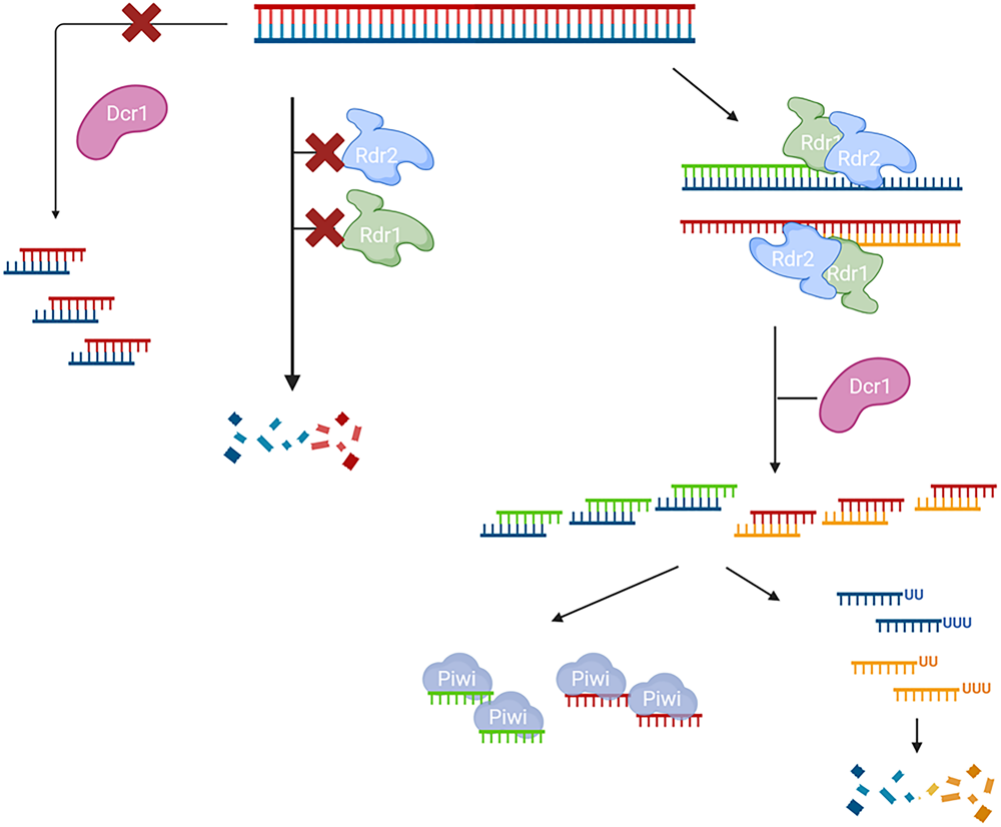
Working Model of RDR-dependent 1^∘^siRNA production after dsRNA feeding. A working model for feeding-induced 1^∘^ siRNA synthesis in *Paramecium*. Exogenous dsRNA cannot be cleaved by Dicer directly (left). In absence of either RDR1 or RDR2, exogenous RNA becomes degraded (middle). In WT cells, both RDRs need to interact to convert dsRNA into new hybrid-dsRNA, which is the Dicer substrate. It is likely that Dicer directly associates with the complex formed by both RDRs. Since siRNA becomes oligouridinylated while antisense siRNAs accumulate, being stabilized in Piwi proteins. (Right) Created in BioRender. Simon, M. (2025) https://BioRender.com/5f9pujd.

Our data provide several new insights that suggest novel mechanistic roles for eukaryotic RDRs. First, RDR1 and RDR2 appear to be required to separate self- from non-self RNA and to convert this exogenous RNA into Dicer-cleavable dsRNA (Fig. 6). It is a striking difference to the literature from other species that Dicer is not the first instance to recognize exogenous dsRNA and, in particular in relation to *C. elegans*, that this RDR function is also capable of converting ssRNA into siRNA^26^. In fact, our data suggest that dsRNA-induced RNAi in *Paramecium* is not triggered by dsRNA itself: dsRNA delivery may simply be more efficient because the trigger is more stable than ssRNA. Even though we can clearly demonstrate RDR activity, we conclude that our data cannot be classified under the term ’RNAi amplification’, as the necessary conditions are not met. In line with the literature, we cannot demonstrate the production of large quantities of 2° siRNAs from mRNA. While there are 2° siRNAs, they are less abundant than the 1° triggers (see Fig. 4C, D and Supplementary Fig. 2B). A new finding is that RDRs copy exogenous RNA at a ratio of 1:1. Again, this does not result in massive amplification, which would result in many more siRNAs from RDR products. Instead, the function of RDRs appears to resemble a RDR-gated 1° siRNA production process more than actual amplification.

We hypothesize that RDR1 and RDR2 work in complexes together with Dicer; complex formation may depend on the C-terminal part of RDRs, as they are necessary for activity. We see differences in RDR2 dependency between ds- and ssRNA feeding, whereas RDR1 is involved in both. This means that both RDRs are not redundant but have specific roles, which is in agreement with previous reports that, for example, RDR2 is involved in transgene-induced silencing, while RDR1 is not^27^.

The complexes in which RDRs associate remain to be characterized. RDRCs (RNA-directed RNA polymerase complexes) in general have been described in many species, however, in particular in association with endogenous RNA. In the related *ciliate Tetrahymena*, Dicer associates with RDR as shown for the production of endogenous siRNAs^28^; In yeast, RDRC is associated with heterochromatin and pericentromeric RNA, and in *C. elegans*, an RDRC has been shown to be necessary for endogenous 26G-RNA synthesis^29^. RDRCs have yet not been reported to act on exogenous templates. It remains unknown how exogenous RNA is recognized, since both RDRs also accept endogenous RNAs and are involved in endogenous siRNA generation^30^.

This new role for RDRs appears to be conserved at least in *Paramecium* spp. as phylogenetic analyses indicate orthologs also in the distant *P. bursaria* (Supplmentary Fig. 9), which agrees with earlier analyses^31^. Since other species’ RDR orthologs cluster mainly due to the organisms source, we cannot rule out the possibility that the role of RDRs in trigger recognition holds true for other unicellular eukaryotes.

What would be the biological role of RDR-mediated non-self RNA recognition and entry into the RNAi pathway? An antiviral mechanism seems logic, but it can to be tested since not a single virus is known to infect *Paramecium*. *Paramecium* in wildlife is known as a host for a great variety of endocytosymbionts, prokaryotes, and eukaryotes^32^. Recent evidence proofs an RNA-RNA interaction of *P. bursaria* with its facultative symbiont, the green algae chlorella, which is maintained in phagosomes: the meaning of siRNA generation from symbiont RNA may not just be in a hijacking mode in which a parasite modulates the host, but in an evolutionary conserved synchronization of metabolic integration and symbiont population control to enable a long term maintenance of the symbiotic interaction^33,34^. RDR activity on exogenous RNA could be seen as an entry control into this mechanism, allowing the host to maintain control, in this case, to prevent Dicer from uncontrolled cleavage of dsRNA or secondary structures in ssRNA.

One would also have to question the general function of 2° siRNAs in protists. In multicellular species, RDR mediated RNAi amplification means the production of large amounts of 2° siRNAs: few trigger molecules in form of 1° siRNAs cleaved from exogenous triggers are sufficient to produce many more 2° siRNAs, for instance in *C. elegans* by 100fold^22^. This makes sense for systemic silencing in order to disseminate an increasing number of siRNAs in multicellular organisms to achieve virus resistance in cells far away from the trigger. A single-celled species would not need such massive amplification, and thus a different mechanistic usage of RDRs seems plausible. Although 2° siRNAs exist outside the feeding region, their abundance is in single digit percentage of 1° siRNAs. This is not in the meaning of amplifying RNAi.

From a biochemical point of view, it remains unknown why two RDRs are necessary for this activity on exogenous RNA. Future studies will need to characterize the RDRC composition and biochemical activity of RDRs. Given that we see differences in dsRNA and ssRNA that feed on the dependency of RDR2 and in reference to studies in *Tetrahymena* indicating several different RDRCs associated with a single RDR^35^, we expect a great complexity of different RDRCs in *Paramecium*.

## Methods

### Cell culture, synchronization, phylogenetics, structural analysis

Wild-type and different mutant strains of *Paramecium tetraurelia* were maintained at 31 ° C in wheat grass powder (WGP) medium supplemented with *Klebsiella planticola* as a food source for culture maintenance or bacteria prepared for feeding experiments, and beta-sitosterol to promote growth as described before^36^. Before experiments, cells were age-synchronized by daily isolation of single cells in fresh WGP medium and incubation at 31 °C for 24 h, repeating isolation for a week. Cells were then incubated for 2 days in fresh medium to induce starvation and thereby autogamy. Autogamy was verified by DAPI staining.

For phylogenetic analysis, amino acids data were aligned with ClustalX and evolutionary history was inferred using the neighbor joining method with bootstraps replicates. Structural alignment of AlphaFold2 predicted structures^37^ was carried out in Colab v1.5.2^38^ and visualized using the Smith-Waterman 3D alignment method^39^ in RSCB Protein Data Bank^40^.

### Heteroduplex design

To distinguish smallRNAs produced directly from the exogenous dsRNA through Dicer cleavage from smallRNAs produced with intermediate RDR activity, a heteroduplex dsRNA was designed. Both strands of the heteroduplex dsRNA harbor a “mismatch area”, a region where mismatches compared to the target mRNA sequence are introduced (compare Fig. 2C and Supplementary Fig. 2A). In the same area, additional mismatches are introduced only to the sense strand of the heteroduplex dsRNA. Following the distribution of the mismatches within sequenced smallRNA reads allows for differentiation between smallRNA reads derived from the exogenously applied heteroduplex dsRNA and reads derived from RDR-converted strands. Sequences of the sense and antisense strand of the heteroduplex dsRNA, as well as the sequence of the *ND169* gene targeted by the heteroduplex dsRNA can be found in Suppl. Table 1.

### Heteroduplex transcription, packaging and delivery

PCR products corresponding to the two heteroduplex RNA strands were created, harboring T7 promoters and terminators for subsequent in vitro transcription. In vitro transcription was carried out by the HiScribe T7 High Yield RNA Synthesis Kit from NEB following the manufacturer’s instructions. Prior to DNAse I treatment and phenol/chloroform extraction, the produced RNA strands were annealed to a heteroduplex dsRNA by heating the reaction to 85 °C for 10 min followed by slow cooling to 4 °C over a period of 45 min.

Annealed heteroduplex dsRNA was mixed in aqueous solution at a 1:2 mass ratio with DEAE dextran 20 (TdB Labs, Uppsala) to facilitate nanoparticle formation using electrostatic interactions. The size of the particles and the net charge were measured using a Zetasizer Ultra (Malvern Panalyitcal) and the particles were visualized by transmission electron microscopy using a JEOL JEM-2100 in bright field mode with a slow-scan charge-coupled device camera at 200 kV operating voltage. To facilitate heteroduplex nanoparticle uptake in *P. tetraurelia*, positively charged particles were adsorbed onto the negatively charged surface of *E. coli* by harvesting a bacterial overnight culture, washing the bacteria twice in 1× PBS and mixing 1 ml of resuspended *E. coli* with 50 μL nanoparticle suspension and incubating the mixture for 30 min at 37 °C under constant shaking. Bacteria were then mixed with 20 ml WGP medium. Paramecia were grown in this medium for 24 h at 27 °C before RNA extraction.

### RNA feeding using *E. coli*

For single strand RNA application, *E. coli* cells of the HT115 DE3 strain (originally described in ref. ^41^) were transformed with a plasmid that harbors a fragment of the *ND169* gene under control of a T7 promoter and terminator. To prepare single-strand RNA (ssRNA) feeding medium, a culture of the transformed *E. coli* was inoculated using an overnight culture and grown to an OD of 0.4 before IPTG was added to induce ssRNA production. ssRNA production was induced for 2.5 h until the bacteria were transferred to WGP medium. Paramecia were cultivated in feeding medium for 48 h at 27 °C before RNA extraction. DsRNA feeding using the same *E. coli* strain was performed, using a *L4440* vector containing a fragment of the *ND169* gene, using the same protocol as explained for ssRNA feeding as established for *Paramecium* before^42^.

### RNA extraction, sRNA-seq, and biochemistry

RNA was extracted from 50.000 cells using TRI Reagent (Sigma-Aldrich). RNA was size-selected using a UREA-MOPS PAGE and enriched for RNA molecules between 18 and 30 nt. Size-selected RNA was used as an input for sRNA library preparation using the NEBNext Small RNA Library Prep Kit for Illumina from NEB according to the manufacturer’s instructions following the size selection by TBE-PAGE protocol. To distinguish sRNAs with different biochemical properties, mainly their 5′-phosphorylation status, size-selected sRNA was treated in different ways to create libraries specific for certain modifications. For 5′-mono-phosphate specific libraries, the NEBNext Small RNA Library Prep Kit for Illumina from NEB, based on classical ligation, was used. For 5′-mono- and tri-phosphate smallRNAs, size-selected RNA was treated with the CapClip enzyme (Cellscript) and then converted into a library as described, or untreated size-selected RNA was subjected to library preparation by using a template switch-based library kit (D-Plex Small RNA-seq Library Prep Kit for Illumina from Hologic Diagenode) as desribed before. For 5′-tri-phosphate specific libraries, size-selected RNA was first Terminato (Epicenter) treated to digest 5′-mono-phosphate RNA. After extraction with acid phenol, the remaining RNA was treated with the CapClip enzyme (Cellscript) before being subjected to the NEBNext Small RNA Library Prep Kit for Illumina from NEB. Successful treatment for biochemical discrimination of RNA was validated by using RNA spike-in oligos with known 5′-phosphorylation status, controlled using a UREA-MOPS PAGE (compare Supplementary Fig. 1A). A DNA spike-in oligo was used as a loading control. Libraries were multiplexed and sequenced on the Illumina NextSeq.

### Data processing and analysis

Reads were de-multiplexed and quality- and adapter-trimming was carried out using the TrimGalore tool, which uses Cutadapt^43,44^. Quality of reads was evaluated using the FastQC/MultiQC tool^45,46^. Only reads between 18 and 30 nucleotides (nt) in length were maintained to ensure presence of at least one mismatch in each read to properly identify the corresponding strand (compare Supplementary Fig. 2A). For heteroduplex analysis, reads were mapped to the two heteroduplex sequences simultaneously, using the Bowtie mapper allowing no mismatches or multi-mapping^47^. Reads mapping on the individual strands of the heteroduplex in either sense or antisense orientation were quantified using in-build Tools of the Geneious Prime 2024.0 Software (https://www.geneious.com). Sense-oriented reads mapping to the sense strand and antisense reads mapping to the antisense strand of the heteroduplex, thereby corresponding to the native orientation of the applied heteroduplex dsRNA in regards of the mismatch positions between the strands, were considered derived from the exogenous dsRNA and labeled as senseHD_sense and antisenseHD_antisense, respectively. Antisense-oriented reads mapping to the sense strand and sense reads mapping to the antisense strand of the heteroduplex, differing from the original orientation of the mismatch positions between the strands, were considered RDR-products and labeled as senseHD_antisense and antisenseHD_sense, respectively.

For analysis of non-templated nucleotides, a modified version of a custom snakemake pipeline was used (https://www.github.com/greenjune-ship-it/untemplated-nucleotides-search). In short, sRNA reads were mapped to the template sequence without allowing mismatches using Bowtie^47^. Then, reads mapped to the sequences were extracted, sense and antisense directed reads were separated and sequence logos for each read length were calculated with the weblogo tool^48^. Reads extracted this way were considered not containing non-templated nucleotides. In a second iteration, reads not mapped were trimmed by removing a single base from the 3′-end using Cutadapt^44^ and mapped to the template sequence again. Reads mapped in this iteration were processed in the same way as described above and were considered to carry a single non-templated nucleotide. This process was repeated a total of four times, allowing for the analysis of up to three non-templated nucleotides. Overlap analysis of siRNAs was performed using the siRNA and piRNA overlap signature prediction tool (ref. ^49^).

### RDR localization

For localization of RDR1 and RDR2, young *Paramecium* cells were transfected by macronuclear injection of a linearized plasmid containing the open reading frame of either RDR fused with an N-terminal GFP. To enhance visualization of RDR localization, GFP-positive cells were used to perform an immunofluorescence assay using anti-GFP antibodies (Roche) using a for *Paramecium* adapted protocol^50^. In short, 10.000 cells were washed once in Volvic water, starved for 30 min, collected in 500 μL and permeabilized for 30 min by adding 500 μL permeabilization buffer (1% formaldehyde, 2.5% Triton X-100, 4% sucrose in 1xPHEM). Following permeabilization, 7 ml fixation buffer (4% formaldehyde, 1.2% Triton X-100, 4% sucrose in 1× PHEM) was added and fixation was carried out for 10 min. Cells were washed twice in blocking solution (2% BSA in TBST), incubated with anti-GFP-antibodies (1:300 in blocking solution) overnight, washed twice with blocking solution, and incubated with secondary antibody (AlexaFluor 555 conjugated F(ab′) (Thermo Fisher) 1:3000 in blocking solution) for 1 h. After a last washing step, cells were mounted onto a cover slip and fluorescent pictures were taken using a fluorecent microscope (Axio Observer, Zeiss). Proper expression of the RDR-GFP fusion proteins were validated by western Blot (compare Supplementary Fig. 10).

### Statistics and reproducibility

For the biochemical analysis of feeding-associated siRNAs, total RNA extracted from a cell culture undergoing dsRNA-feeding was used. The very same total RNA sample was split in four aliquots, each aliquot being treated to obtain the four different 5′-phosphorylation-dependent libraries displayed in Fig. 1. Heteroduplex dsRNA containing nanoparticles were produced and applied four times, representing four true biological replicates used for analysis of heteroduplex-derived 1° siRNAs as well as non-templated nucleotide analysis. The fourth batch of heteroduplex nanoparticles was also applied to the described mutant strains, allowing comparison of siRNAs of this wildtype replicate with siRNAs produced from mutant strains. ssRNA feeding was carried out in biological duplicates for mutant strains and one replicate for wildtype cells simultaneously, allowing for comparison between the wildtype and mutant replicates. Mean value and standard deviation of the two mutant biological replicates were calculated to compare siRNA abundance between samples. Three additional biological wildtype replicates were obtained later, but fed with a different batch of induced bacteria, which is why these replicates were only considered for read length distribution of produced siRNAs.

### Reporting summary

Further information on research design is available in the Nature Portfolio Reporting Summary linked to this article.

## Supplementary information

Supplementary Information

Description of Additional Supplementary Files

Supplementary Data 1

Reporting Summary

## Data Availability

All sequencing data generated in this study are available in the ArrayExpress database (http://www.ebi.ac.uk/arrayexpress) under accession number MTAB-14931. Maps of plasmids generated in this study are available under 10.5281/zenodo.17815827^51^. Numerical sources data of figures presented in this study are provided in Supplementary Data 1. All other data will be available from the corresponding author upon reasonable request.

## Code availability

For analysis of non-templated nucleotides, a custom snakemake pipeline using established tools was used. An in-depth explanation of the workflow is provided in the methods section, including the specific tools used in each step. The base version of the pipeline is available via GitHub (https://github.com/greenjune-ship-it/untemplated-nucleotides-search). A modified snakefile realizing the split of sense and antisense mapping reads as well as an additional script normalizing the created weblogos to the *paramecium* genome base composition, as used in the final analysis of the data in this manuscript, are provided under 10.5281/zenodo.17896661^52^. Specific requirements for using the pipeline, including software versions, are provided in GitHub as well as the Zenodo repository.
