# Supplementary material for: Exogenous dsRNA made accessible to Dicer by two eukaryotic RNA-dependent RNA polymerases in Paramecium tetraurelia
Source: Commun Biol. 2026 Jan 8;9:167. doi: 10.1038/s42003-025-09443-4 (PMC12876857; doi:10.1038/s42003-025-09443-4)
Supplement: Supplementary file 1 — Supplementary Information [file 42003_2025_9443_MOESM1_ESM.pdf]

Supplemental Material

Exogenous dsRNA made accessible to Dicer by two eukaryotic RNA-dependent RNA polymerases in *Paramecium tetraurelia*

Marcello Pirritano, Johannes Buescher, Pauline Staubach, Thorsten Tacken, Yulia Yakovleva, Mark Sabura, Kristela Shehu, Sören Franzenburg, Marc Schneider, Martin Simon

Suppl. Fig. 1

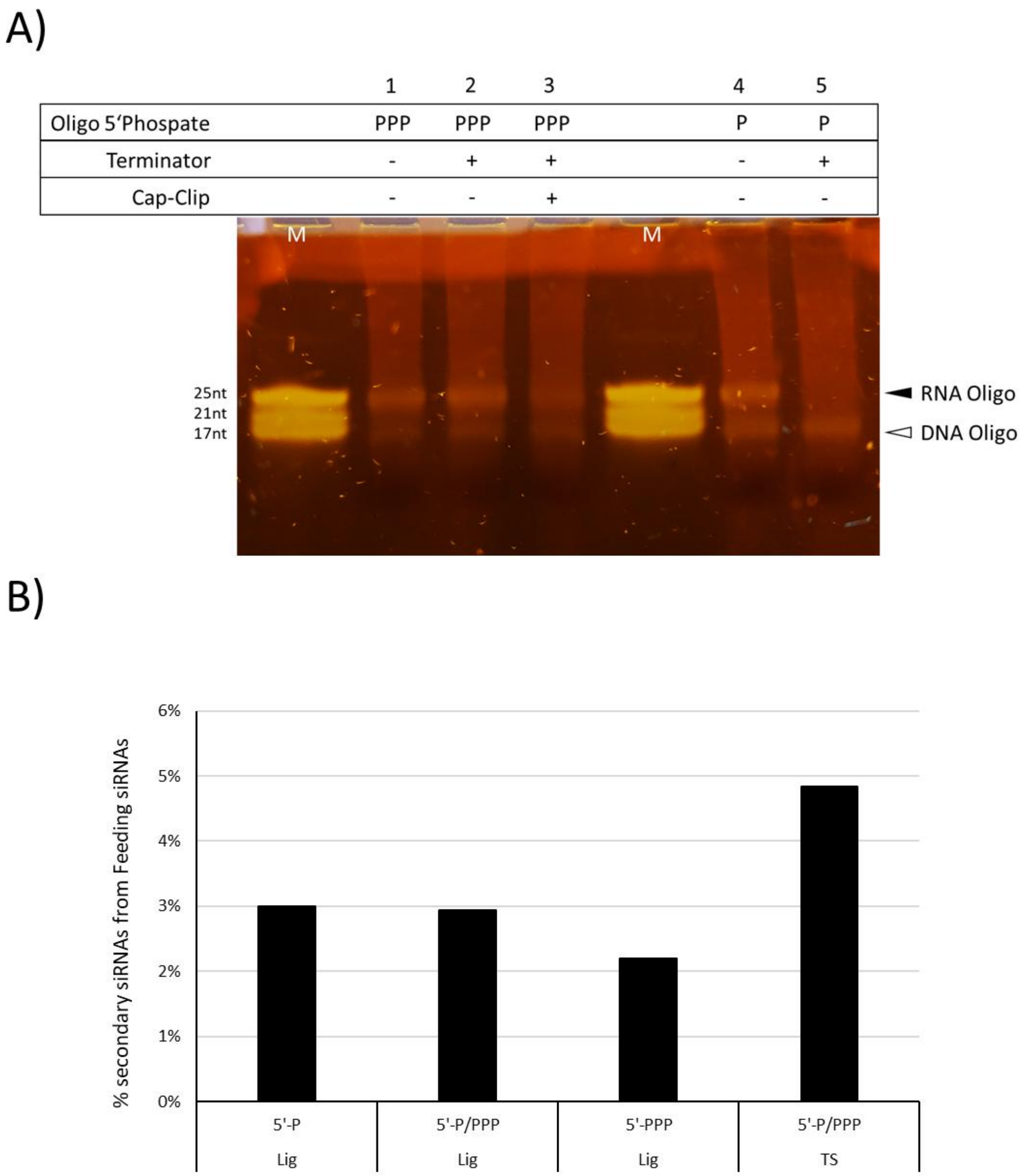

A) Analysis of control RNA oligos (5'-tri-phosphate (left) are 5'-mono-phosphate (right) as indicated in above line) during biochemical treatment of RNA samples. A DNA oligo resistant to all treatments was used as a loading control. Analysis on the right shows treatment with Terminator 5´-monophosphate specific exonuclease (lane 2) and subsequent treatment with CapClip pyrophosphatase to create 5´-monophosphate ends at remaining RNAs suitable for ligation (lane3). Lane 1 shows RNA sample with oligos without any treatment. Lane 4 shows the RNA sample with control oligos whithout and lane 5 with Terminator treatment. All treatments of RNA samples were carried out in duplicate, one with and one without control oligos. Parallel samples without control oligos were sequenced.

B) Ratio of 2° siRNAs in relation to total feeding associated smallRNA reads within the different libraries discriminating between biochemical status of the 5' phosphorylation. (Lig – ligation based library, TS- template switch based library)

Suppl. Fig. 2

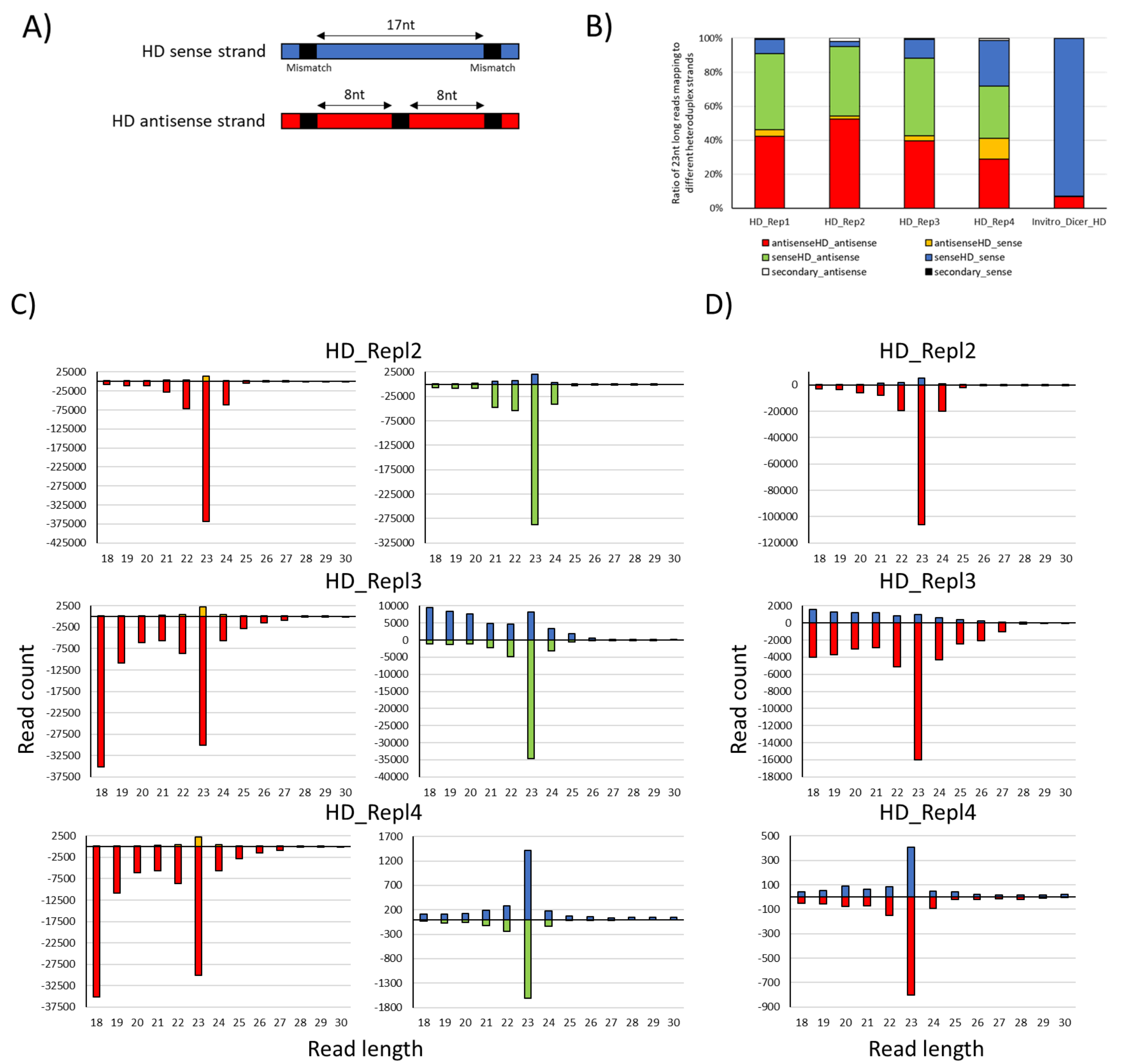

Application of heteroduplex dsRNA loaded Dextran nanoparticles

A) Schematic overview of mismatch distribution within the mismatch area of the heteroduplex dsRNA. Mismatches (black bars) and the distance between the mismatches are indicated in the sense (blue) and the antisense (red) strand of the heteroduplex.

B) Composition of 23nt siRNA reads mapping to different strands. Displayed are four biological replicates of cells fed with Heteroduplex dsRNA in addition to heteroduplex dsRNA that has been processed by an *in vitro* Dicer system instead of being applied to cells as a negative control.

C) Read length distribution of reads mapping to the four possible heteroduplex dsRNA derived strands. Read count of reads corresponding to sense and antisense strands of the original strands (blue and red, respectively) and to the RDR-dependent products (green and orange) are shown.

D) Read length distribution of reads mapping to the sense and antisense strand of the applied heteroduplex dsRNA outside of the mismatch area. Distinguishing RDR-dependent products from exogenous strands is not possible here due to the lack of mismatches. Read count of reads corresponding to sense and antisense strands (blue and red, respectively) are shown.

Suppl. Fig. 3

A)

B)

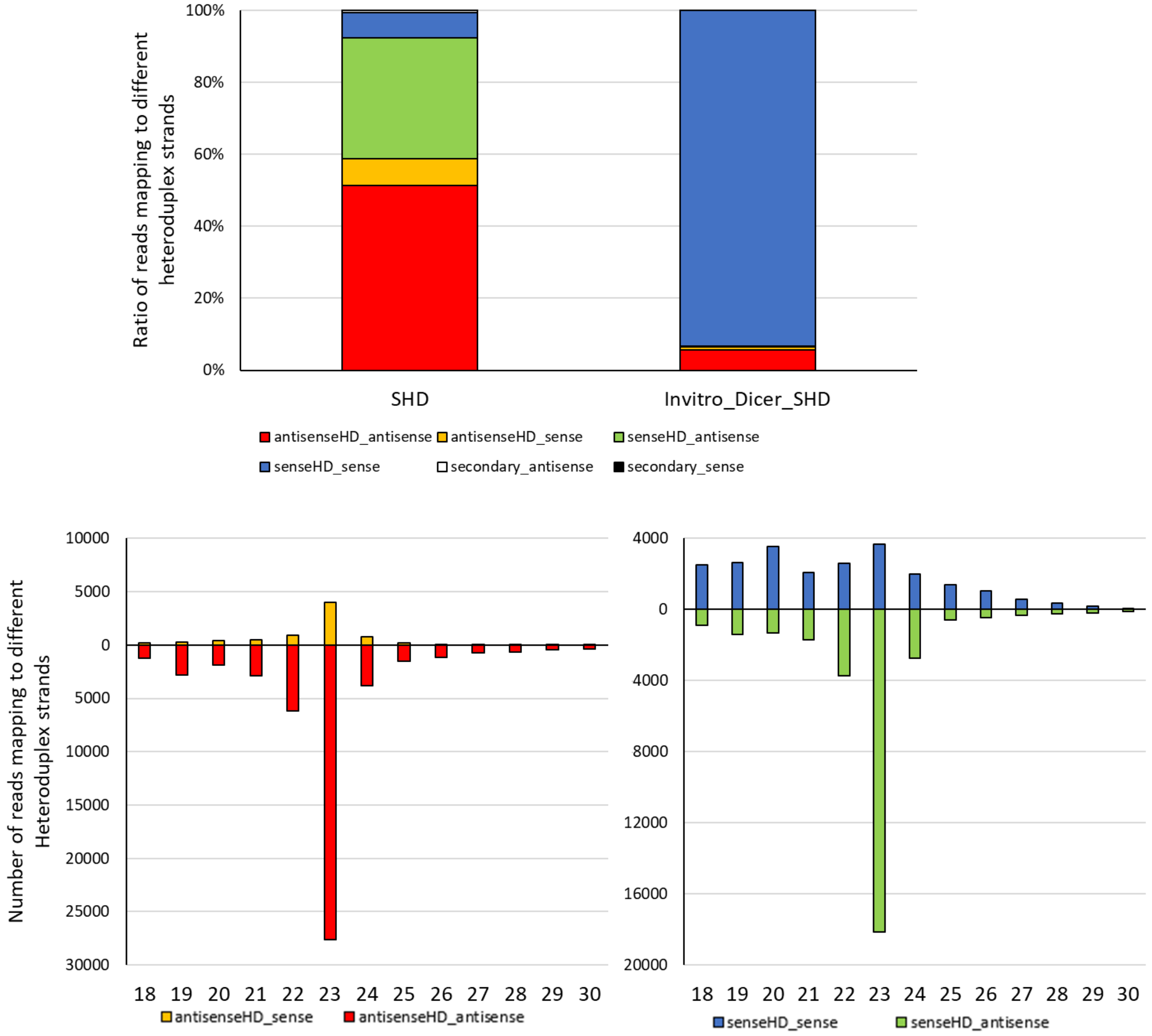

Switch heteroduplex application.

A) Ratio of 23nt siRNA reads mapping to different switch heteroduplex-associated sequences. Cells were fed with switch heteroduplex dsRNA which has the additional mismatches to dissect between both strands not in the antisense but the sense strand (blue) (Ext Fig. 2A). The same dsRNA has also been subjected for *in vitro* Dicer digestion.

B) Read length distribution of small RNA reads mapping to sense and antisense strands of the switch heteroduplex.

Suppl. Fig. 4

A)

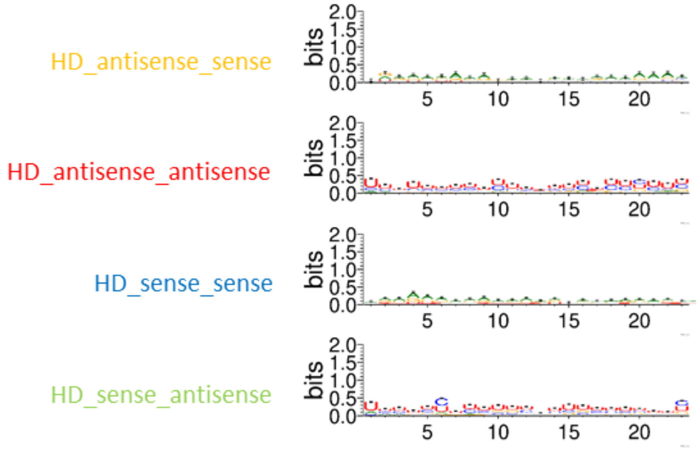

B)

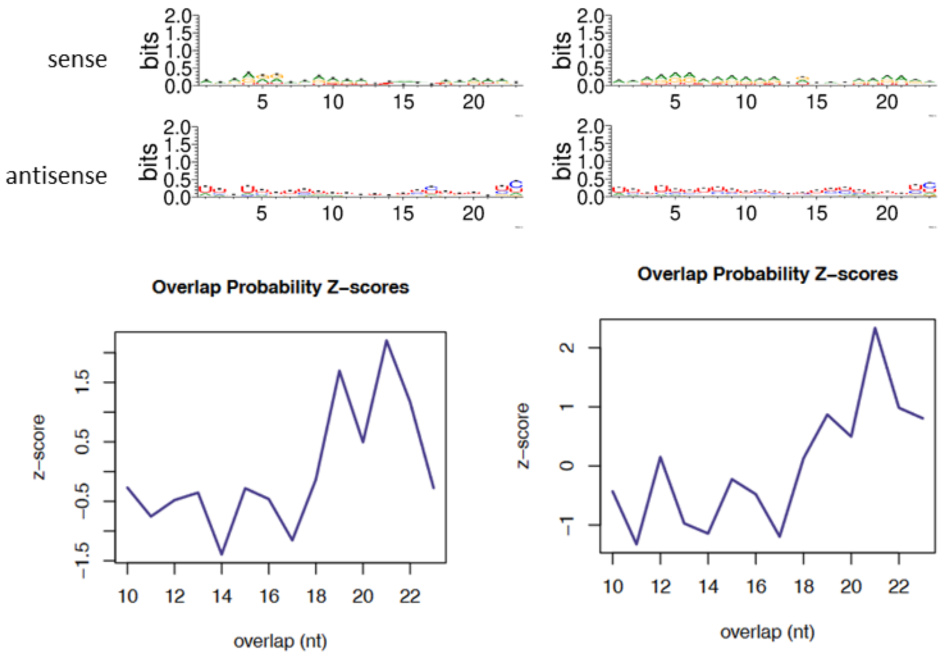

A) Sequence Logo of 23nt siRNAs produced from Heteroduplex dsRNA feeding. Displayed are the generated logos for each of the four possible Heteroduplex strands, identified using the incorporated mismatches.

B) Sequence Logo of 23nt siRNAs produced from ordinary bacteria-based dsRNA feeding. Displayed are the generated logos for sense and antisense orientated siRNAs. In addition to sequence logo generation, overlap analysis of 23nt siRNA reads was performed. Two replicates of dsRNA feeding are displayed.

Suppl. Fig. 5

A)

RdRP1

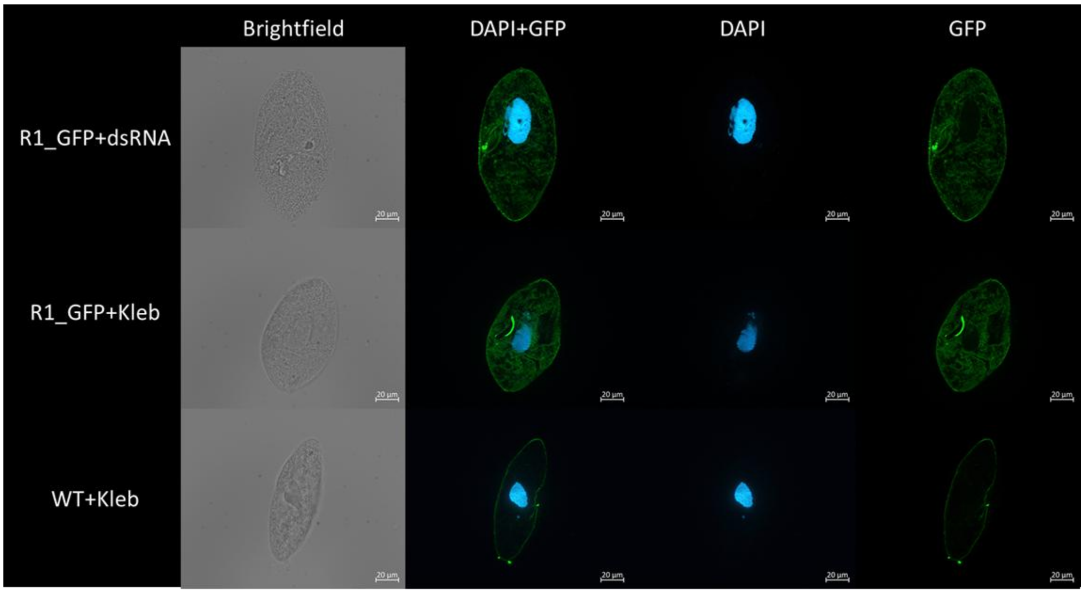

B)

RdRP2

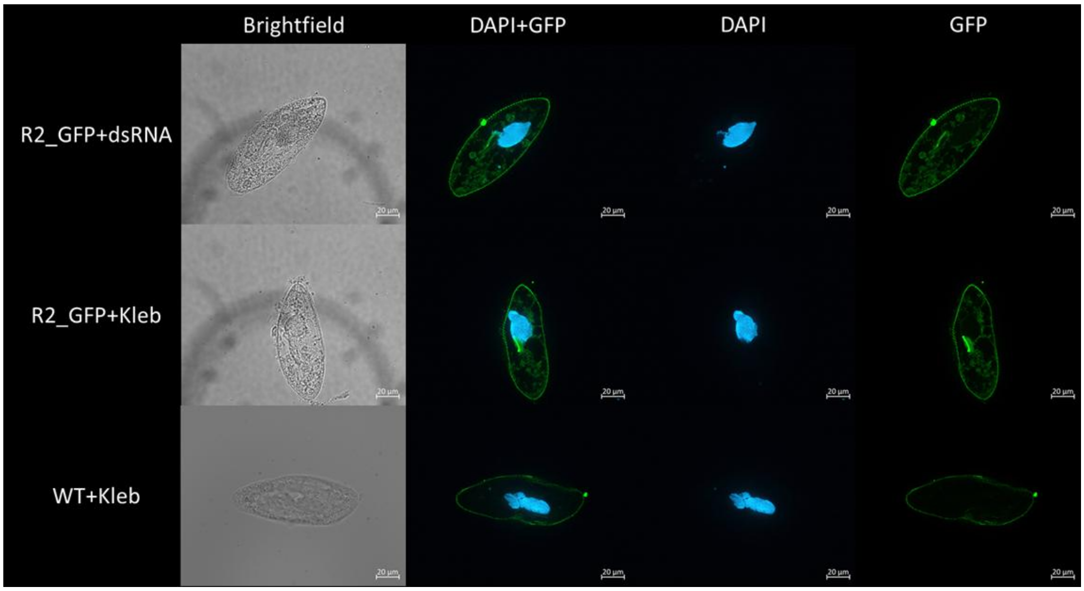

Immunofluorescence assay localization of N-terminal GFP-tagged RdRP1 and RdRP2.

Fluorescence signals were improved by anti-GFP antibodies with DAPI as counterstaining showing the macronucleus. RDR1 (A) and RDR2 (B). Brightfield images for displayed cells are provided. Localization of both RDRs was performed for both, the presence of dsRNA within food bacteria (+dsRNA) and bacteria without (+Kleb).

Suppl. Fig. 6

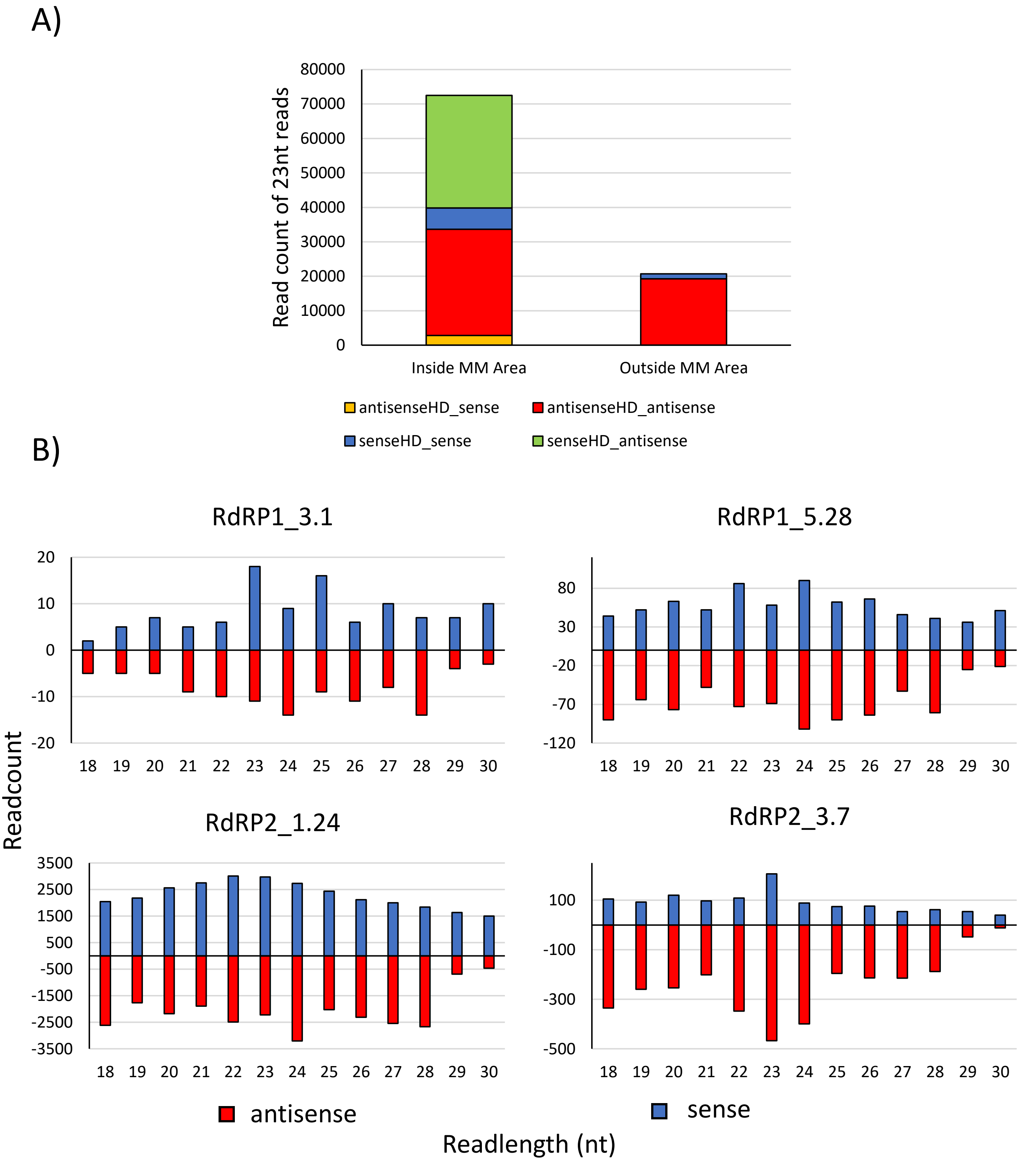

A) Comparison of 23nt siRNA read abundance of reads mapping to the mismatch are (MM Area) and outside of the MM area of HD_Repl1. Compared are read counts of the areas displayed in figure 2D/E.

B)Read length distribution of reads mapping to sense (blue) and antisense (red) Strands of the Heteroduplex dsRNA outside of the mismatch area. Distinguishing of Exogenous RNA strands and RDR-produced strands is not possible in this region, due To lack of mismatches.

Suppl. Fig. 7

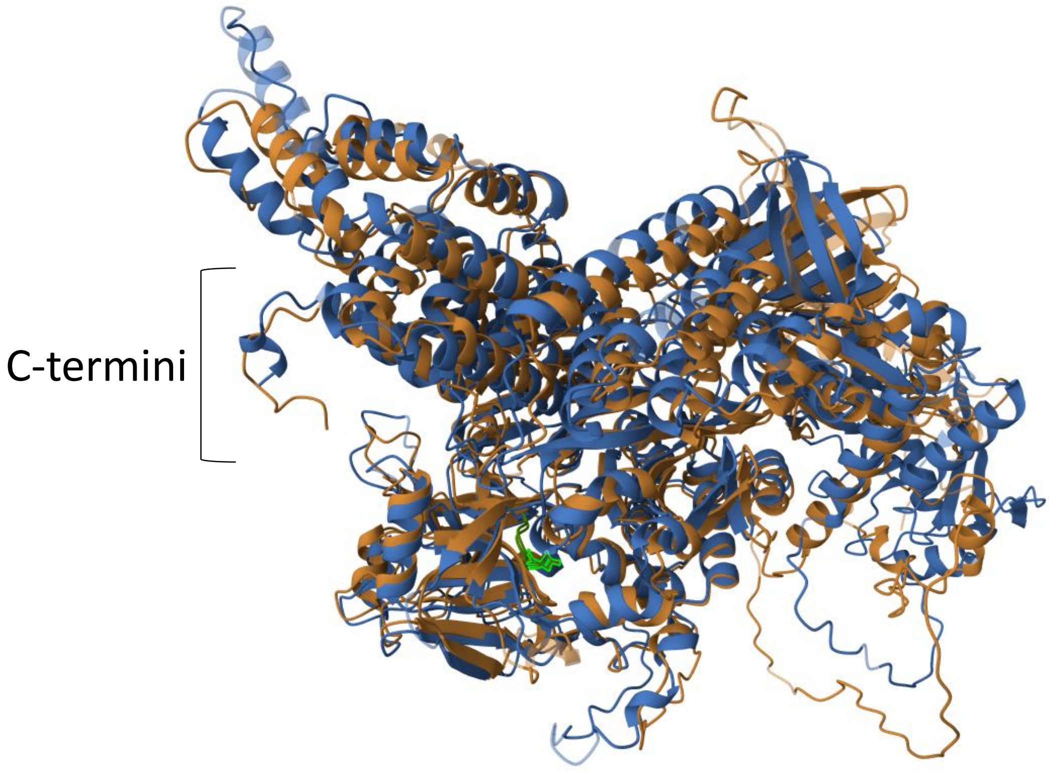

Structural alignement of Alpha Fold2 predicted structures of RDR1 (brown) and RDR2 (blue). C-terminal parts are indicated on the left and the catalytic DLDGD motif of both RDRs is indicated in green.

Suppl. Fig 8

A)

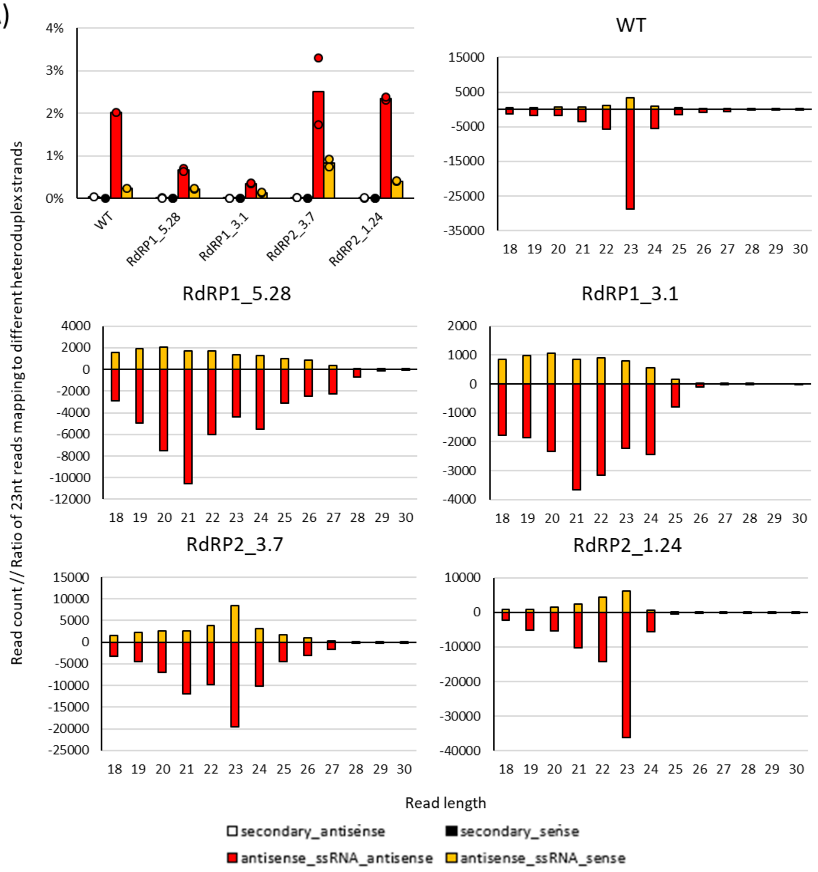

B)

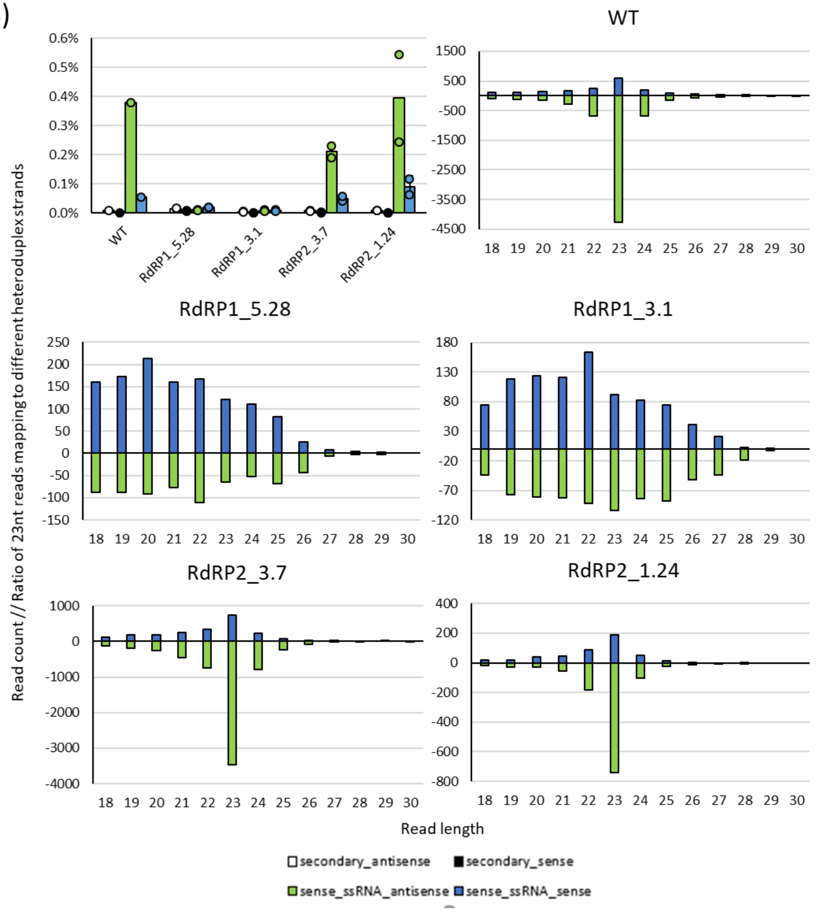

Single strand RNA feeding.

Mapping statistics and length distributions of different cell lines fed with antisense (A) or sense (B) orientated ssRNA.

Mapping statistics (first Figure in each panel) show the ratio of 23nt siRNA reads mapping to different heteroduplex related sequences in relation to total 23nt reads for representative replicates. Bars represent the mean value while individual data points represent value of replicates. Read length distributions are shown for wildtype or mutant strains, respectively.

Suppl. Fig. 9

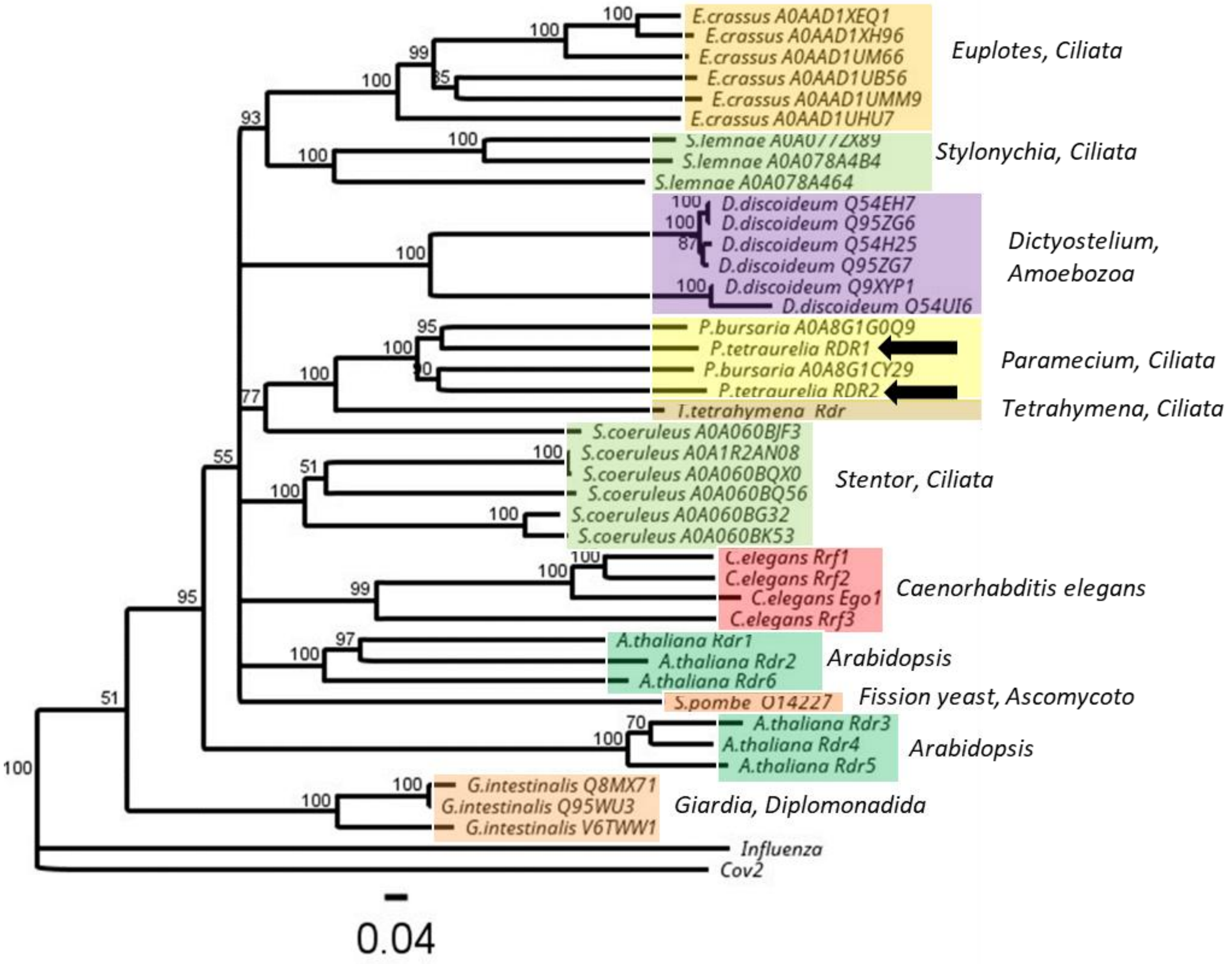

Neighbor joining consensus tree of RDR proteins rooted to the Sars-Cov-2 RDR. Supportvalues are given at the nodes.
For undesribed RDRs, UniProt Acc Nrs are indicated next to the organisms. Arrows indicate RDR1/2 from *P.tetraurelia*.

Suppl. Fig. 10

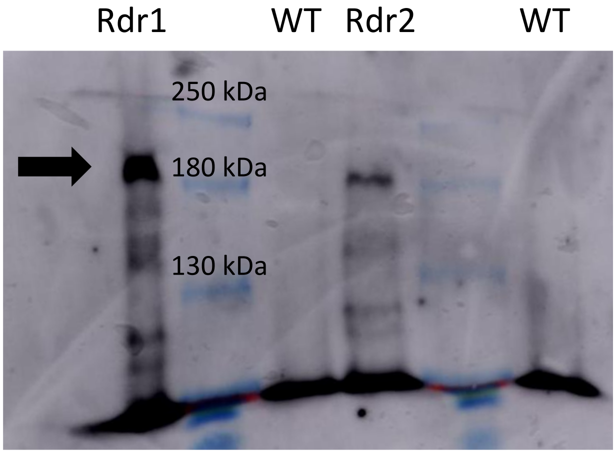

Western Blot of total protein from cells transformed with GFP-fusion constructs of Rdr1, Rdr2, or untransformed wildtype cells (WT). Detection of the expressed GFP-fusion protein was carried out using a custom αGFP-antibody. The black arrow indicates the full-length GFP-fusion protein, which is 190 kDa (for Rdr1-GFP) and 184 kDa (for Rdr2-GFP) in size.

Suppl. Table 1

Sequences of sense and antisense strand of the heteroduplex dsRNA as well as the ND169 target sequence. Mismatch area of the heteroduplex dsRNA is labeled in yellow. Mismatches of both, sense and antisense, strand compared to the target gene sequence are color-coded in red, while mismatches distinguishing sense and antisense strand of the heteroduplex dsRNA are colored in blue.

>Heteroduplex_senseStrand

GAGAAGAATCCCCTCAGGAAAATTAAGAGTAAGAGGATTCATCCAATGCTTCAGGATAAGGAGCCAACAACA
GGATCTAAGTGAAAAAGGAGAAAAAGTAGAAGAGCAATTCTATATTTATAAGCTAGAAATTGTGTAAATACTT
GCTGAAGAAACAGCAGTGCCAGAGGAAGAATAAGTGCCTGAAGCATTATAAAAAGAAGTGTAGGAAGAAAT
AGTTGAATAGGGAGAAAAAGACGTGGAATATGTGATTGAGACATCCAAAGAACATAAGGTAGATATTCGTAA
AATTCACGTGGAGAAGTAGAAGAAGAAGAAGGTTTACAAGGAGGAAATTGAGAATAAAAAATATAAGGATC
AAGGAGATAGAGATATAGAAGGAGATTTAGAAACAGATTATTACAAAGATTA

>Heteroduplex_antisenseStrand

TAATCTTTGTAATAATCTGTTTCTAAATCTCCTTCTATATCTCTATCTCCTTGATCCTTATATTTTTTATTCTCAATT
TTCTCCTTGTAAACCTTCTCCTTCTTCTACTTCTCCATGTGAATTTTACGAATATTTACCTTATGTTCTTTGGGTGT
CTCAATCACATATTTCACGTCTTTTTCTCCCTGTTCAACTATTTCTTCCTGCACTTCTTTTTATAATGTTTCAGGCA
CTTATTCTTTCTCTGGCACTGCTGTTTTTTCAGCAAGTATTTACATAATTTCTAGCTTATAAACATAGAATTGCTC
TTCTATTTTTTCTCCTTTTTCACCTAGATCCTGTTGTTGGCTCCTTATCCTGAAGCATTGGATGAATCCTCTTACTC
TTAATTTTCCTGAGGGGATTCTTCTC

>ND169_targetSequence

GAGAAGAATCCCCTCAGGAAAATTAAGAGTAAGAGGATTCATCCAATGCTTCAGGATAAGGAGCCAACAACA
GGATCTAAGTGAAAAAAGAGAAAAAGTAGAAGAGTAATTCTATATTTATAAGTTAGAAATTGTGTAAATATTT
GCTGAAGAAACAGCAATGCCAGAGGAAGAATAAATGCCTGAAGCATTATAAGAAGAAGTGTAGGAAGAAGT
AGTTGAATAGGGAGAAGAAGACGTGGAATATGTGTTTGAGACATCCAAAGAATATAAGGTAGATATTCGTGA
AATTCACGTGGAGAAGGAGAAGAAGAAGAAGGTTGACAAGGAGGAAATTGAGGATAAAAAATATAAGGATC
AAGGAGATAGAGATATAGAAGGAGATTTAGAAACAGATTATTACAAAGATTA
