# Supplementary material for: Exogenous dsRNA made accessible to Dicer by two eukaryotic RNA-dependent RNA polymerases in Paramecium tetraurelia
Source: Commun Biol. 2026 Jan 8;9:167. doi: 10.1038/s42003-025-09443-4 (PMC12876857; doi:10.1038/s42003-025-09443-4)
Supplement: Supplementary file 2 — Description of Additional Supplementary Files [file 42003_2025_9443_MOESM2_ESM.pdf]

Description of Additional Supplementary Files

File name: Supplementary Data 1

Description: Numerical source data
